# Detecting partial premature ovulation during follicular aspiration compromises the quantity, but not the quality, of the oocytes retrieved in stimulated in vitro fertilization (IVF) cycles

**DOI:** 10.5935/1518-0557.20240003

**Published:** 2024

**Authors:** Víctor Hugo Gómez, Cristina Rodríguez-Varela, Elena Labarta, Ernesto Bosch

**Affiliations:** 1 IVI RMA Valencia. Human Reproduction Department. Plaza Policía Local, 3. 46015 Valencia, Spain; 2 IVI Foundation - IIS La Fe. Research Department. Avenida de Fernando Abril Martorell, 106. 46026 Valencia, Spain

**Keywords:** premature ovulation, IVF, oocyte retrieval, follicular aspiration, oocyte quality, embryo quality

## Abstract

**Objective:**

To analyze if partial premature ovulation (PPO) detection during oocyte pick-up (OPU) impairs the quality of the retrieved oocyte cohort.

**Methods:**

The PPO concept refers to the situation when premature ovulation happens only in some of the follicles and it is detected during OPU. This study constitutes a retrospective analysis performed in an infertility clinic (Spain) during 2016-2021 with patients undergoing OPU after controlled ovarian hyperstimulation for an *in vitro* fertilization (IVF) treatment. Study code: 2110-VLC-091-VG, registered on December 9 2021. Data from women with PPO (n=111) were compared to a matched control sample of cycles without PPO (n=333) at a proportion of 1:3.

**Results:**

Cycles were matched for age, body mass index (BMI), treatment year, embryo genetic analysis and stimulation protocol type. The mean numbers of oocytes (6.1 *vs*. 11.2), mature oocytes (4.7 *vs*. 8.8), correctly fertilized oocytes (3.6 vs. 6.6) and top-quality blastocysts (0.9 *vs*. 1.8) were significantly lower in the PPO group than the nonPPO group (*p*<0.05). However, maturation, fertilization, top-quality blastocyst and pregnancy rates were statistically comparable among groups (*p*>0.05).

**Conclusions:**

Cycles with PPO have fewer available oocytes and, thus, fewer available embryos for transfer, al though their quality is intact, and still offer chances of pregnancy in these cases. Hence cycle cancellation may not be worth associated money, time and morale losses once PPO is detected.

## INTRODUCTION

Follicular aspiration in *in vitro* fertilization (IVF) treatments is scheduled 36 hours (h) after triggering with an ovulation inductor. This trigger induces an *in vivo* resumption of the first meiosis in those oocytes present inside preovulatory follicles (Abbara *et al*., 2018). Currently, pituitary suppressors are often prescribed to avoid spontaneous ovulation prior to trigger administration (Diedrich *et al*., 1994). In some cases however, a dominant follicle partially ruptures prematurely and prior to the scheduled hour for follicular aspiration.

In our case, we refer to “partial premature ovulation” (PPO) when this phenomenon is observed by ultrasound while the oocyte pick-up (OPU) procedure is being performed. We thus called it if there was a soon-to-be-formed corpus luteum structure or a low follicular count than expected in the last ultrasound performed prior to OPU, with both conditions in association or not with free fluid in the pouch of Douglas.

In some cases, a prematurely ruptured dominant follicle does not mean that the oocyte enclosed inside it has been expelled, which has been previously described in the literature (Craft *et al*., 1980; Stanger & Yovich, 1984). Furthermore Teramoto *et al.* (2019) not only demonstrated that oocytes can be retrieved from this type of follicles, but also proved their competence.

However, one of the main concerns about this phenomenon is the competence of the whole retrieved oocyte cohort after it is detected. It would be logical to think that the oocytes retrieved from these ruptured follicles, if obtained, may be in a different maturity stage and may, thus, be less competent. Moreover, partial ovulation may have induced a dominance phenomenon in the rest of the cohort, which leads them to the atresia, or at least to an impaired final maturation process (Son *et al*., 2011).

Partial ovulation has not been extensively studied in the literature. In this retrospective study, we aim to describe our own experience in an infertility clinic in Spain regarding such cases. We analyze the frequency of this event, the number of retrieved oocytes once detected, and the maturation, fertilization, blastocyst formation and pregnancy rates related to these oocytes. We also aim to assess if there is any potential early predictor of PPO that could provide us with an early indication of its occurrence.

## MATERIALS AND METHODS

### Design and setting

A retrospective study performed at an infertility clinic in Spain between 2016 and 2021. It includes all the patients submitted to a follicular aspiration procedure after a controlled ovarian stimulation protocol for IVF treatment.

### Study population

Female patients having undergone follicular aspiration for IVF treatment after controlled ovarian stimulation treatment and during the study period, regardless of semen origin. Mixed cycles with both aspirated and thawed oocytes, as well as oocyte donation cycles and oocyte vitrification cycles, were excluded from the analysis.

### Ovarian stimulation protocol

Ovarian stimulation was performed following the routine clinical practice in IVIRMA Valencia, as described elsewhere (Giles *et al*., 2021; Melo *et al*., 2009). Ovulation induction was triggered when 3 or more follicles ≥18 mm were confirmed by transvaginal ultrasound, using the gonadotropin releasing hormone (GnRH) agonist (Decapeptyl^®^, Ipsen Pharma, France), the human chorionic gonadotropin (hCG) (Ovitrelle^®^, Merck & Co., Inc, USA) or through the combined action of both of them.

Follicular aspiration was carried out under sedation and transvaginally ultrasound-guided 36 hours after administration of the ovulation trigger.

### Operating room procedures

Patients with premature partial ovulation (PPO) were detected by the clinician during the follicular aspiration procedure and registered in the patient’s clinical history. The concept of PPO refers to the event in which the extrusion of the oocyte by the follicle, or at least the beginning of the ovulation process, happens earlier than expected according to protocol (prior to 36 hours after ovulation induction). In addition, we call it partial ovulation because it occurs in only part of the follicles, not the entire cohort. Diagnosis was based on the presence of soon-to-be-formed corpus luteum structure/s and/or a lower follicular count than expected compared to the last ultrasound performed prior to OPU, and in association or not with free fluid in the pouch of Douglas.

Around 80% of cases were evaluated by the same clinician because all the OPUs performed in the clinic are done by the same gynecologist. Only the OPUs scheduled at weekends and during holidays were done by the gynecologist in charge of on-call duty.

There was no traceability for the follicle of origin of the retrieved oocytes. Hence, unlike Teramoto *et al*., 2019, we cannot affirm if the retrieved oocytes came from the ruptured follicles in PPO cycles.

### IVF lab procedures

The oocytes retrieved during follicular aspiration were denuded 4 h after the procedure. After denudation, mature oocytes (metaphase II) were those with an extruded first polar body and no germinal vesicle visible in the cytoplasm. The aspiration rate was defined as the number of retrieved oocytes per number of follicles in the last ultrasound performed before pick-up. The maturation rate was defined as the number of mature oocytes per total number of oocytes.

Only the mature oocytes were fertilized by intracytoplasmic sperm injection (ICSI). The fertilization rate was defined by the number of correctly fertilized oocytes per total number of fertilized oocytes. Correctly fertilized oocytes were those with two pronuclei and two polar bodies 17 h after fertilization.

The correctly fertilized oocytes were cultured *in vitro* until the blastocyst stage on day 5 or 6 of development. Embryo quality was classified as A, B or C following the classification of the Spanish Association for the Study of Reproduction Biology (ASEBIR) (Pons, 2015). Embryo quality was assessed by one of the 15 senior embryologists who make up the team of embryologists in the clinic.

The top-quality blastocyst rate was defined by the number of top-quality blastocysts per total number of available blastocysts. The top-quality blastocysts were those classified as A or B. In the pre-implantational genetic testing for aneuploidies (PGT-A) cycles, the top-quality blastocysts were considered only those classified as A or B, which were also euploid.

Embryo transfer (ET) was performed on day 5 or 6 of development by senior gynecologists with transabdominal ultrasound guidance.

### Pregnancy outcomes

The biochemical pregnancy outcome was determined by a positive β-hCG test (serum levels of β-hCG >10 IU/ml 11 days after ET). Clinical pregnancy was defined as the presence of at least one gestational sac upon ultrasound. Ongoing pregnancy was defined as the presence of at least one viable fetus beyond week 12, and live birth when pregnancy resulted in at least one live born neonate. The biochemical miscarriage rate was defined as a positive b-hCG test with no evidence for a gestational sac and clinical miscarriage after confirming an intrauterine gestational sac. Ectopic pregnancy was defined as a gestational sac located outside the uterine cavity. The cumulative ongoing pregnancy rate after the first, second, third and fourth ET attempt was also calculated.

### Statistical analysis

The data from the women with PPO were compared to a matched control sample of cycles without partial ovulation during the same study period. A proportion of 1:3 (PPO: nonPPO) was used. Matching was performed with RStudio through the library “MatchIt” following the nearest neighbor method on the propensity score, which selects patients with the most similarity in the variables used for matching. Cycles were matched for age, BMI, treatment year, performed embryo genetic analysis and stimulation protocol type.

The numeric variables are shown as mean±standard deviation and were compared by an ANOVA test. For the numeric variables with a negative homogeneity of variances test, a Mann-Whitney test was performed instead. The categorical variables were shown by proportion and compared by the Chi-square test.

A power analysis was run to compare the mean number of top-quality blastocysts (0.982), and the mean top-quality blastocyst rate (0.487), between the PPO and nonPPO groups to indicate the power of each comparison given our sample size. The power analysis was calculated for these two variables because they account for the final main IVF treatment outcome before ET. A power analysis was also performed to compare pregnancy rates to indicate the statistical power of pregnancy outcomes given the small sample size and the few ETs that derived from the cycles included in this study. It showed powers of 0.298 for the comparison of the biochemical pregnancy rate, 0.166 for the clinical pregnancy rate and 0.050 for the comparison of the ongoing pregnancy rate.

## RESULTS

### Descriptive analysis

During the study period, 8801 ovarian stimulation cycles for IVF treatment with fresh own oocytes were performed. PPO was detected in 117 of these cycles, which led to the occurrence of < 2% in our study population.

The cycles with no aspirated oocyte were excluded from the subsequent analysis to obtain a fair overview of embryological results. For this reason, 204 cycles of the control group and two cycles from the PPO were excluded. There were four women with PPO in which OPU was not performed once ovulation had been detected (final n=8591).

**Figure 1:**
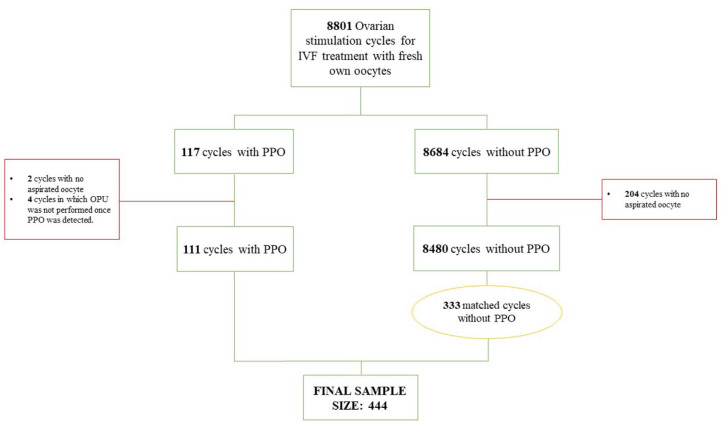
Flow chart of the IVF cycles included. PPO = premature partial ovulation. OPU = oocyte pick-up.

In this population, 111 PPO cases were detected. The data from these cycles were compared to a matched control sample of cycles without PPO (n=333) (PPO 1:3 nonPPO) (Figure 1).

No statistically significant difference in cycle characteristics was found, nor in the ovarian stimulation protocol prior to OPU (Table 1) between the PPO and nonPPO groups.

**Table 1:** Baseline and cycle characteristics of the overall population (n=444) and each group (control *vs*. PPO group). The overall data are shown in the first column, while columns 2 and 3 contain the data that refer to each group and their comparison. The numeric variables are shown as mean±standard deviation. A *p* value refers to the comparison using an ANOVA test. ^MW^Mann-Whitney test due to a negative homogeneity of variances test; non-parametric data are shown by median±interquartile range. The categorical variables are shown as proportion. A *p* value refers to the comparison using the Chi-square test. ^*^p≤0.05 was considered statistically significant. BMI = body mass index; AMH = antimullerian hormone; PGT-A = pre-implantational genetic testing for aneuploidies. PPO = partial premature ovulation. FSH = follicle stimulating hormone. hMG = human menopausal gonadotropin. hCG = human chorionic gonadotropin. GnRh-a = gonadotropin releasing hormone agonist. E2 = estradiol. P4= progesterone. IQR = interquartile range.

	OVERALL N=444	PPO GROUP N=111	CONTROL GROUP N=333	*p* value
Age	37.8±3.8	37.7±3.3	37.9±4.0	0.684
BMI	23.9±4.1	23.9±4.0	23.9±4.1	0.928
AMH	2.2±2.1	1.9±2.7	2.3±1.9	0.190
Semen origin Partner semen Donated semen	348/404 (86.1%) 56/404 (13.9%)	64/71 (90.1%) 7/71 (9.9%)	284 (85.3%) 49 (14.7%)	0.282
PGT-A Yes No	248 (55.9%) 196 (44.1%)	58 (52.3%) 53 (47.7%)	190 (57.1%) 143 (42.9%)	0.377
Treatment year 2016 2017 2018 2019 2020 2021	70 (15.8%) 100 (22.5%) 68 (15.3%) 93 (20.9%) 82 (18.5%) 31 (7.0%)	16 (14.4%) 35 (31.5%) 11 (9.9%) 21 (18.9%) 16 (14.4%) 12 (10.8%)	54 (16.2%) 65 (19.5%) 57 (17.1%) 72 (21.6%) 66 (19.8%) 19 (5.7%)	0.023^*^
Cause of infertility Advanced maternal age Male factor Low reserve/low response Karyotype alteration/genetic disease Endometriosis Polycystic ovarian syndrome Infertility of unknown origin Other	195 (43.9%) 129 (29.1%) 32 (7.2%) 14 (3.2%) 12 (2.7%) 11 (2.5%) 22 (5.0%) 29 (8.7%)	46 (41.4%) 33 (29.7%) 12 (10.8%) 6 (5.4%) 4 (3.6%) 2 (1.8%) 5 (4.5%) 3 (2.7%)	149 (44.7%) 96 (28.8%) 20 (6.0%) 8 (2.4%) 8 (2.4%) 9 (2.7%) 17 (5.1%) 26 (7.8%)	0.166
Ovarian stimulation Days of stimulation Total gonadotropin dose (UI)	10.8±2.0 2986.2±1275.4	11.1±2.2 3137.7±1376.1	10.7±1.9 2953.7±1252.6	0.138 0.277
Stimulation protocol Antagonists Agonists Progestagens Clomiphene citrate Other Type of trigger hCG GnRH agonist hCG & GnRH agonist	335 (75.5%) 5 (1.1%) 60 (13.5%) 24 (5.4%) 20 (4.5%) 88 (19.8%) 265 (59.7%) 91 (20.5%)	83 (74.8%) 2 (1.8%) 15 (13.5%) 6 (5.4%) 5 (4.5%) 13 (11.7%) 80 (72.1%) 18 (16.2%)	2 52 (75.7%) 3 (0.9%) 45 (13.5%) 18 (5.4%) 15 (4.5%) 75 (22.5%) 185 (55.6%) 73 (21.9%)	0.923 0.007^*^
Triggering day E2 levels (pg/ml) P4 levels (ng/ml) No. follicles	2108.8±1417.9 0.57 (IQR 0.74) 11.9±5.8	1808.2±1286.9 0.82 (IQR 1.32) 11.0±6.0	2174.5±1438.5 0.55 (IQR 0.64) 12.1±5.7	0.048^*^ 0.015^MW*^ 0.180
Triggering day: Right Ovary No. follicles No. follicles≥10mm No. follicles≥13mm Biggest follicle (mm)	5.9±3.4 5.5±3.4 4.8±3.2 19.0±2.7	5.5±3.5 5.3±3.6 4.8±3.4 19.1±2.9	6.0±3.4 5.5±3.4 4.8±3.2 19.0±2.6	0.276 0.562 0.990 0.719
Triggering day: Left Ovary No. follicles No. follicles≥10mm No. follicles≥13mm Biggest follicle (mm)	5.9±3.0 5.4±3.2 4.8±3.0 18.9±2.9	5.4±3.1 5.1±3.2 4.7±3.1 19.2±3.3	6.0±3.0 5.4±3.2 4.8±3.0 18.9±2.8	0.192 0.396 0.759 0.475

### Oocyte quantity and quality

The variables related to oocyte quantity were significantly lower in the PPO patients than in the nonPPO patients. The variables related to oocyte quality were similar among groups (Table 2).

**Table 2:** The oocyte quantity and quality variables in the overall population (n=444) and each group (PPO vs. control group). The numeric variables are shown as mean ± standard deviation. A *p* value refers to the comparison using an ANOVA test. ^MW^Mann-Whitney Test due to a negative homogeneity of variances test; non-parametric data are shown by median±interquartile range. The categorical variables are shown as proportion. A p value refers to the comparison using the Chi-square test. ^*^p≤0.05 was considered statistically significant. PPO = partial premature ovulation. IQR = interquartile range.

	OVERALL N=444	PPO GROUP N=111	CONTROL GROUP; N=333	*p* value
OOCYTE QUANTITY Aspiration rate No. oocytes No. mature oocytes No. correctly fertilized oocytes No. top-quality blastocysts	84.8% 8.0 (IQR 9.0) 6.0 (IQR 8.0) 4.5 (IQR 6.0) 1.0 (IQR 2.0)	61.8% 5.0 (IQR 5.0) 4.0 (IQR 4.0) 3.0 (IQR 4.0) 0.0 (IQR 1.0)	89.6% 10.0 (IQR 9.5) 8.0 (IQR 8.0) 6.0 (IQR 6.0) 1.0 (IQR 2.0)	< 0.001^*^ <0.001MW^*^ <0.001 MW^*^ <0.001MW^*^ <0.001 MW^*^
OOCYTE QUALITY Maturation rate Fertilization rate Top-quality blastocyst rate	80.5% 73.7% 27.3%	80.1% 75.9% 21.9 %	80.6% 72.9% 29.0%	0.816 0.295 0.054

### Embryo transfer and pregnancy results

In all, 154 ETs of 95 patients were performed. Of these, 17 ETs accounted for PPO cycles from 12 patients and 137 ETs for the control group cycles of 83 patients.

ETs were fresh in 35.7% of cases (55) and frozen in the remaining 64.3% (99). Frozen ETs were performed in the natural cycle context in 16.2% of cases (16) and in 83.8% (83) of cases. A PGT-A analysis was performed in 4.0% of the frozen ET cases (4).

There were no significant differences among groups for any variable related to ET and pregnancy results (Table 3).

**Table 3:** Main characteristics of the performed ETs in the overall population (n=444) and the comparison between the PPO and control groups. The numeric variables are shown as mean ± standard deviation. A p value refers to the comparison using an ANOVA test. The categorical variables are shown by proportion. A *p* value refers to the comparison using the Chi-square test. ^*^p ≤ 0.05 was considered statistically significant. ET = embryo transfer; PPO = partial premature ovulation.

	OVERALL N=444	PPO GROUP N=111	CONTROL GROUP; N=333	p value
DAY OF ET Day 5 Day 6	83.8% 16.2%	88.2% 11.8%	83.2% 16.8%	0.596
NO. TRANSFERRED EMBRYOS 1 2	81.8% 18.2%	88.2% 11.8%	81.0% 19.0%	0.467
EMBRYO QUALITY A B C	25.7% 55.9% 18.4%	18.8% 50.0% 31.3%	26.5% 56.6% 16.9%	0.360
PREGNANCY RATES Biochemical pregnancy (%) Clinical pregnancy (%) Ongoing pregnancy (%) Live birth (%) Biochemical miscarriage (%) Clinical miscarriage (%) Cumulative ongoing pregnancy (%) After first ET attempt After second ET attempt After third ET attempt After fourth ET attempt	66.9% 59.7% 46.8% 44.6% 9.7% 21.7% 27.3% (42/154) 39.6% (61/154) 44.2% (68/154) 46.1% (71/154)	82.4% 70.6% 47.1% 43.8% 14.3% 33.3% 29.4% (5/17) 41.2% (7/17) 41.2% (7/17) 47.1% (8/17)	65.0% 58.4% 46.7% 44.7% 9.0% 20.0% 27.0% (37/137) 39.4% (54/137) 44.5% (61/137) 46.0% (63/137)	0.151 0.334 0.979 0.943 0.534 0.296 0.850 0.718 0.840 0.841
NO. ET ATTEMPTS PER PATIENT	1.62±0.96	1.42±0.90	1.65±0.97	0.432
NO. ET ATTEMPTS UNTIL ONGOING PREGNANCY OR LIVE BIRTH PER PATIENT	1.34±0.61	1.29±0.49	1.35±0.63	0.787

### Potential parameters related to partial premature ovulation

Table 1 shows that the only significantly different variables in the PPO and control groups were the type of trigger, as well as E2 and P4 levels on the triggering day. Cycles in the PPO group have higher E2 and P4 levels on the triggering day, and triggered with higher frequency using the GnRH agonist.

The adjusted binary logistic regression model for the phenomenon of PPO is shown in Table 4.

**Table 4:** Adjusted binary logistic regression model for the phenomenon of premature partial ovulation (PPO). Confounding factors in the first half table are serum E2 and P4 levels on the triggering day, as well as triggering with GnRH-a *vs*. hCG or hCG and GnRH-a; in the second half table are serum E2 levels, P4 levels ≥ 1.5 ng/ml *vs*. levels below this cut-off and triggering with GnRh-a. ^*^p≤0.05 was considered statistically significant. hCG = human chorionic gonadotropin. GnRH-a = gonadotropin releasing hormone agonist. E2 = estradiol. P4 = progesterone. CI = confidence interval.

	Adjusted	95% CI	p value
Odds Ratio (OR)			
E2 levels triggering day	**1.000**	0.999 - 1.000	0.002^*^
P4 levels triggering day	**1.940**	1.297 - 2.902	0.001^*^
GnRH-a (vs. hCG or hCG+GnRH-a)	**2.624**	1.293 - 5.325	0.008^*^
	**Adjusted** **Odds Ratio (OR)**	**95% CI**	**p value**
E2 levels triggering day	**1.000**	0.999 - 1.000	0.002^*^
P4 ≥ 1.5 ng/ml on the triggering day (vs. P4 < 1.5 ng/ml)	**4.095**	1.812 - 9.256	0.001^*^
GnRH-a (vs. hCG or hCG+GnRH-a)	**2.458**	1.199 - 5.040	0.014^*^

Regarding the P4 levels, those of 13.6% of the overall population were over the cut-off point of 1.5ng/ml (Bosch *et al*., 2010) on the triggering day. Of these, 38.5% exhibited PPO, while 61.5% were from the control group (*p*<0.001). In the PPO group, 28.8% of patients had serum P4 levels ≥1.5ng/ml *vs.* 10.2% in the control group (*p*<0.001). The adjusted binary logistic regression model for the phenomenon of PPO taking into consideration the variable serum P4 levels on the triggering day as categorical (yes *vs*. no) is shown in Table 4.

The P4 levels above the cut-off point were related to: a significantly larger mean number of follicles in the last ultrasound performed before OPU (14.4±5.8 *vs.* 11.6±5.4 in the group of patients with P4 below 1.5ng/ml; *p*=0.004); a significantly bigger mean number of oocytes retrieved after OPU (14.7±11.3 *vs.* 9.8±6.5 in the patients with P4 below 1.5ng/ml; *p*<0.001).

## DISCUSSION

PPO significantly reduces the quantity, but not the quality, of the oocytes available for IVF treatment. Hence the cycles with PPO will have fewer available oocytes and, thus, fewer available embryos for transfer, but their quality will remain intact.

To our knowledge, this is the first study to analyze the impact of PPO on the quantity and quality of the whole oocyte cohort in IVF treatments. This phenomenon has been previously addressed in the literature (Craft *et al*., 1980; Stanger & Yovich, 1984), and the competence of the oocytes retrieved from these prematurely ruptured follicles has already been proven (Teramoto *et al*., 2019). However, the aim of the present study was to analyze the competence of the whole retrieved oocyte cohort to shed more light on what was already described by Teramoto *et al*. (2019).

PPO is not a very frequent phenomenon in IVF treatments (below 2% based on our data). Nevertheless, our results may help with not only clinical decision making, but also clinicians to orientate and inform their patients about their chances with the IVF cycle once this phenomenon has been detected.

Given the low PPO frequency in our population, we performed the comparison of oocyte quantity and quality between the PPO IVF cycles and a control group of patients without PPO, but matched to the PPO group for age, BMI, treatment year, performed embryo genetic analysis and stimulation protocol type at a proportion of 1:3. This type of analysis may avoid any bias regarding the huge sample size difference between the PPO and nonPPO groups. In contrast, and consequently, the main limitation of this study is its small sample size.

Our results clearly show a significant drop in both the recovery rate and the final total number of oocytes, mature oocytes, correctly fertilized oocytes and top-quality blastocysts (Table 2). Therefore, PPO significantly reduces oocyte quantity and, hence, the final number of usable blastocysts in that cycle. Indeed PPO reduces the recovery rate beyond the minimum threshold taken in the literature as the optimal recovery rate, which is around 75-85% of expected oocytes (El-Shawarby *et al*., 2004; Braga *et al*., 2020).

In contrast, these data suggest that PPO has no significant impact on oocyte quality, as shown by similar the maturation, fertilization and top-quality blastocyst rates (Table 2). The embryos from the PPO and nonPPO cycles have similar embryo quality rates according to the classification of the Spanish Association for the Study of Reproduction Biology (ASEBIR) (Table 3). Besides, the embryos from the PPO group went to the blastocyst stage at a similar ratio to the control embryos, which also reinforces their similar quality (Table 3). So the fewer oocytes recovered after PPO have exactly the same quality as in the nonPPO cycles.

Furthermore, PPO occurrence does not seem to affect pregnancy rates (Table 3). More importantly, the mean number of ET attempts until ongoing pregnancy or live birth was similar among groups (Table 3). However, these comparisons in pregnancy outcomes do not have enough statistical power (0.298 for biochemical pregnancy, 0.166 for clinical pregnancy and 0.050 for ongoing pregnancy) to detect significant differences, which is probably due to the few ETs performed in both groups, especially in the PPO group.

Hence if we clinicians detect PPO occurrence, but we think it is not worth canceling the cycle despite fewer oocytes, we at least know that their quality will not be lost. Nevertheless, if they are implemented in routine clinical practice, the parameters for detecting PPO should be better defined to unify criteria.

However, can we predict PPO occurrence in IVF treatments and, thus, act beforehand? Our results show that serum E2 and P4 levels on the triggering day, as well as the type of trigger, could be potential markers of PPO (Table 1). Cycles with PPO had significantly higher E2 and P4 levels on the triggering day, and triggered with higher frequency using the GnRH agonist. After having adjusted for confounding factors, the three of them showed a significant correlation with PPO (Table 4).

Regarding serum P4 levels ≥1.5ng/ml, which seem to exert the highest effect, it is true that a significantly higher proportion of PPO patients had P4 levels above this cut-off point (28.8% in the PPO group *vs.* 10.2% in the control group; *p*<0.001). Levels above this threshold point have been related to lower pregnancy rates in fresh ETs (Bosch *et al*., 2010), which was, thus, the cut-off point used in this analysis.

However, the proportion of patients with P4 levels exceeding this threshold on the triggering day had significantly lower PPO (38.5% in the PPO group *vs.* 61.5% in the control group; *p*<0.001). In addition, P4 levels ≥1.5ng/ml were related to significantly more follicles in the last ultrasound prior to OPU (14.4±5.8 *vs.* 11.6±5.4 in the group of patients with P4 below 1.5 ng/ml; *p*=0.004) and to significantly more retrieved oocytes (14.7±11.3 *vs.* 9.8±6.5 in the patients with P4 below 1.5 ng/ml; *p*=0.018). This scenario is completely the opposite of what our data suggest for the patients with PPO.

High serum P4 levels on the triggering day might indicate the onset of premature ovulation risk and, hence, its correlation to PPO occurrence during OPU. Indeed it has been suggested that an initial serum P4 rise in the late follicular phase might be the physiological trigger of the ovulatory gonadotropins surge in humans prior to luteinizing hormone (LH) and estradiol peaks (Dozortsev & Diamond, 2020). Nevertheless, our data cannot ensure PPO occurrence after having detected P4 levels above this cut-off point on the triggering day.

In any event, main limitations of the present study include its retrospective design and its limited sample size, derived from the low occurrence of PPO in IVF treatments. Thus, it is possible that potential differences in oocyte quality couldn’t be detected due to the insufficient study power of this analysis. In addition, pregnancy outcomes cannot be firmly compared regarding the small number of embryo transfers included. Therefore, results from this study should be treated with caution.

## CONCLUSION

PPO is a very uncommon phenomenon in controlled ovarian hyperstimulation IVF treatments. Its occurrence significantly reduces the quantity, but not the quality, of the oocytes available for IVF treatment and, thus, still offers chances of pregnancy. Serum P4 levels above the cut-off point of 1.5ng/mL on the triggering day may suggest a higher risk of PPO, but its predictive value has not been confirmed. Therefore, cycle cancellation may not be worth associated losses of money, time and morale once detected.

## References

[r1] Abbara A, Clarke SA, Dhillo WS. (2018). Novel Concepts for Inducing Final Oocyte Maturation in In Vitro
Fertilization Treatment. Endocr Rev.

[r2] Bosch E, Labarta E, Crespo J, Simón C, Remohí J, Jenkins J, Pellicer A. (2010). Circulating progesterone levels and ongoing pregnancy rates in
controlled ovarian stimulation cycles for in vitro fertilization: analysis
of over 4000 cycles. Hum Reprod.

[r3] Braga DPAF, Zanetti BF, Setti AS, Iaconelli A (2020). Borges E Jr. Immature oocyte incidence: Contributing factors and
effects on mature sibling oocytes in intracytoplasmic sperm injection
cycles. JBRA Assist Reprod.

[r4] Craft I, Shelton K, Yovich J, Smith D. (1980). Ovum retention in the human. Fertil Steril.

[r5] Diedrich K, Diedrich C, Santos E, Zoll C, al-Hasani S, Reissmann T, Krebs D, Klingmüller D (1994). Suppression of the endogenous luteinizing hormone surge by the
gonadotrophin-releasing hormone antagonist Cetrorelix during ovarian
stimulation. Hum Reprod.

[r6] Dozortsev DI, Diamond MP. (2020). Luteinizing hormone-independent rise of progesterone as the
physiological trigger of the ovulatory gonadotropins surge in the
human. Fertil Steril.

[r7] El-Shawarby S, Margara R, Trew G, Lavery S. (2004). A review of complications following transvaginal oocyte retrieval
for in-vitro fertilization. Hum Fertil (Camb).

[r8] Giles J, Alama P, Gamiz P, Vidal C, Badia P, Pellicer A, Bosch E. (2021). Medroxyprogesterone acetate is a useful alternative to a
gonadotropin-releasing hormone antagonist in oocyte donation: a randomized,
controlled trial. Fertil Steril.

[r9] Melo M, Busso CE, Bellver J, Alama P, Garrido N, Meseguer M, Pellicer A, Remohí J. (2009). GnRH agonist versus recombinant HCG in an oocyte donation
programme: a randomized, prospective, controlled, assessor-blind
study. Reprod Biomed Online.

[r10] Pons MC., Cuadernos de Embriología Clínica (2015). Criterios ASEBIR de Valoración Morfológica de Ovocitos,
Embriones Tempranos y Blastocistos Humanos.

[r11] Son WY, Das M, Shalom-Paz E, Holzer H. (2011). Mechanisms of follicle selection and development. Minerva Ginecol.

[r12] Stanger JD, Yovich JL. (1984). Failure of human oocyte release at ovulation. Fertil Steril.

[r13] Teramoto S, Osada H, Shozu M. (2019). Prematurely ruptured dominant follicles often retain competent
oocytes in infertile women. Sci Rep.

